# Retinal Microaneurysms Detection Using Gradient Vector Analysis and Class Imbalance Classification

**DOI:** 10.1371/journal.pone.0161556

**Published:** 2016-08-26

**Authors:** Baisheng Dai, Xiangqian Wu, Wei Bu

**Affiliations:** 1 School of Computer Science and Technology, Harbin Institute of Technology, Harbin, China; 2 Department of New Media Technologies and Arts, Harbin Institute of Technology, Harbin, China; Northwestern University Feinberg School of Medicine, UNITED STATES

## Abstract

Retinal microaneurysms (MAs) are the earliest clinically observable lesions of diabetic retinopathy. Reliable automated MAs detection is thus critical for early diagnosis of diabetic retinopathy. This paper proposes a novel method for the automated MAs detection in color fundus images based on gradient vector analysis and class imbalance classification, which is composed of two stages, i.e. candidate MAs extraction and classification. In the first stage, a candidate MAs extraction algorithm is devised by analyzing the gradient field of the image, in which a multi-scale log condition number map is computed based on the gradient vectors for vessel removal, and then the candidate MAs are localized according to the second order directional derivatives computed in different directions. Due to the complexity of fundus image, besides a small number of true MAs, there are also a large amount of non-MAs in the extracted candidates. Classifying the true MAs and the non-MAs is an extremely class imbalanced classification problem. Therefore, in the second stage, several types of features including geometry, contrast, intensity, edge, texture, region descriptors and other features are extracted from the candidate MAs and a class imbalance classifier, i.e., RUSBoost, is trained for the MAs classification. With the Retinopathy Online Challenge (ROC) criterion, the proposed method achieves an average sensitivity of 0.433 at 1/8, 1/4, 1/2, 1, 2, 4 and 8 false positives per image on the ROC database, which is comparable with the state-of-the-art approaches, and 0.321 on the DiaRetDB1 V2.1 database, which outperforms the state-of-the-art approaches.

## Introduction

Diabetic retinopathy (DR) is the commonest complication of diabetes and one of the major causes of blindness. The early diagnosis and treatment of DR are very important to prevent vision impairment. The microaneurysms (MAs) on retina are the small saccular bulges in the walls of retinal capillary vessels [1] and generally appear near to the macula [2]. MAs are the first sign of DR [3]. According to the Early Treatment Diabetic Retinopathy Study (ETDRS) [4], the presence of only even 1 or 2 MAs shows the symptom of the mild non-proliferative diabetic retinopathy (i.e., ETDRS level 20). The more MAs, the higher risk of the progression of retinopathy [5, 6].

However, screening of MAs is usually performed manually by ophthalmologist through visual inspection of the color fundus image [5–7], which is a time-consuming, repetitive, tiring, subjective and error-prone process. Therefore, it is necessary to investigate automated MAs detection in color fundus image.

**Fig 1:**
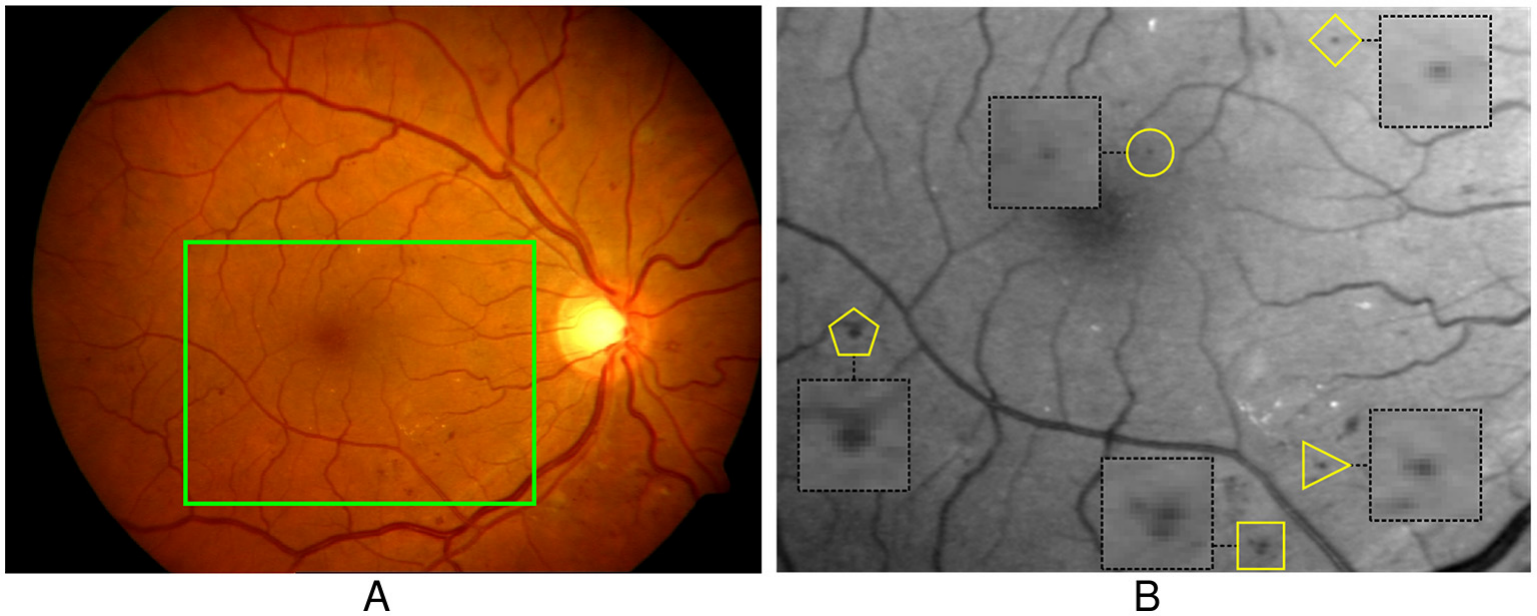
An example of color fundus image containing MAs. (A) The color fundus image. (B) The corresponding enlarged part of (A) in green channel with indicated MAs (diamond: regular MA, circle: subtle MA, triangle: irregular MA, square: clustered MAs, pentagon: MA close to vessel). Reprinted from [8] under a CC BY license, with permission from Dr. Yalin Zheng, original copyright 2012.

MAs always appear as dark red and small round spots in color fundus image. Fig 1 shows a color fundus image containing MAs and a corresponding enlarged part in green channel with some MAs indicated by yellow markers. As shown in Fig 1B, some of MAs are difficult to be found out and separated from the background noises (e.g., the subtle one indicated by the circle). In addition, some MAs may have an irregular shape (e.g., the pedunculated one indicated by the triangle), cluster together (e.g., the clustered ones indicated by the square) or close to vessels (e.g., the one indicated by the pentagon). Thus, automated MAs detection in the color fundus image is a challenging task. In general, there are two stages for automated MAs detection, i.e. candidate MAs *extraction* and *classification*. In the first stage, the candidate MAs are extracted, and in the second stage, the true MAs are identified from candidates by a classifier with a set of extracted features.

For candidate MAs extraction, most of the existing works could be roughly categorized into the matched filter based, the morphology based and the other approaches. In the matched filter based approaches, some special filters were designed to discriminate the MAs from other structures. Quellec et al. [9] and Zhang et al. [10] modeled the MAs with 2D Gaussian functions, and detected the MAs by using template matching [9] or multi-scale correlation filtering [10]. Giancardo et al. [11] used Radon transformation to extract the Gaussian-like circular MAs. Hatanaka et al. [12] proposed a double-ring filter to detect MAs by comparing the intensities between different circular regions. Matched filter based approaches [9–12] worked well when the shapes of MAs are similar with the shape of the filters, but failed to extract some irregular MAs (e.g., MAs with saccular, fusiform, or pedunculated shape [13]), clustered MAs or MAs close to vessels. In morphology based approaches, some morphology characteristics of MAs were used to extract the candidate MAs. Fleming et al. [14] and Ram et al. [15] detected MAs based on the morphological top-hat operation with different linear structural elements. Walter et al. [16] assumed that the diameters of MAs are always smaller than a threshold and proposed a morphological diameter closing operation to extract MAs. Rosas-Romero et al. [17] extracted MAs by using the bottom-hat and hit-or-miss transformations. Morphology based approaches can effectively extract the MAs whose shapes and sizes are similar with those of the structural elements. However, since the shapes and the sizes of different MAs vary largely in fundus images, it is very difficult to define a set of morphological features to characterize and detect all MAs. In the other approaches, such as in [18], a pixel classification followed the morphological operation was used to determine the candidate MAs. In [19], a moat operator was first applied to enhance the edge of candidate MAs, and a recursive region growing was then extracted the area of candidate MAs. It is noted that any true MA lost in this stage cannot be retrieved in the next stage. Hence, to improve the sensitivity of automated MAs detection, it is important to extract as many true MAs as possible in this stage.

Due to the complexity of fundus image, besides the true MAs, the extracted candidate MAs also include a large amount of non-MAs. In candidate MAs classification stage, Fleming et al. [14] defined three types of features for each candidate, i.e., size, intensity and vesselness Boolean features, and then trained a *k*NN classifier to identify true MAs. A sensitivity of 0.54 at the level of 10 false positives per image (FPs/I) was reported on a private dataset. Niemeijer et al. [18] exploited shape, intensity and texture features for each candidate and also trained a *k*NN classifier to recognize the true MAs. A CPM score of 0.395 (competition performance metric, i.e., an average sensitivity at a set of particular false positives per image 1/8, 1/4, 1/2, 1, 2, 4 and 8 FPs/I) was achieved on the ROC database [3]. Giancardo et al. [20] trained a SVM classifier to identify true MAs with a set of features extracted from Radon space and obtained a CPM score of 0.375 on the ROC database. Hatanaka et al. [12] utilized shape, color, statistic and some filter response features to train an ANN for identifying true MAs from non-MAs and obtained a sensitivity of 0.68 at the level of 15 FPs/I on 25 images of ROC training dataset. Lazar et al. [21] proposed a set of intensity profile features and applied a naïve Bayes classifier in candidate MAs classification. A CPM score of 0.423 was achieved on the ROC database. In [22], Fegyver applied a set of features based on gradient directions and lengths and trained a naïve Bayes classifier to identify true MAs. The CPM score of this method is 0.422 on the ROC database. These traditional classifiers work well when the samples of different classes are balanced. However, in the extracted candidate MAs, the number of non-MAs is always more tremendous than the number of true ones. The ratio between the non-MAs and the true MAs is very high, e.g., this ratio reported in [18] was close to 50:1 (14591 non-MAs and 315 true MAs). That is, the true and the non-MAs are extremely imbalanced and those traditional classifiers may not work well for this extremely class imbalanced problem. Class-imbalanced classification should be introduced for MA classification.

Hatanaka et al. [12] limited the maximum number of the candidate MAs in each image, and Lazar et al. [21] manually selected the negative samples (non-MAs) to construct a training set, in which the true MAs and the non-MAs classes are not too class-imbalanced. However, limiting the number of candidates may reduce the sensitivity of MAs detection, and the manually selection of samples from about ten thousand non-MAs is very time consuming and subjective, and lose much information of training samples. In [16], Walter et al. used a Bayesian risk minimization rule to classify the true MAs, where a misclassification cost parameter was introduced to alleviate the problem of class imbalance. But the specific cost information is rarely available, and the method obtained similar results with the *k*NN classifier as reported by the authors [16]. Séoud et al. [23] utilized a Random Forest classifier to overcome the problem of imbalanced training data. However, this classifier may not work well with highly imbalanced data as reported in [24].

Recently, Antal et al. [25] presented an ensemble approach to detect MAs, which selected five image preprocessing operations and five candidate extractors [10, 16, 26–28], to form 25 〈preprocessing operation, candidate extractor〉 pairs with 2^25^ possible combinations. The final MAs were detected by the fusion of the MA candidates which were extracted by the individual pairs in the optimal ensemble. This ensemble strategy has outperformed most existing individual approaches on ROC database [3], in which almost all of the small dark spots in the images are MAs. Actually, in fundus images, besides MAs, there are other objects which are also shown as small dark spots, e.g. the small hemorrhages and scars left after PRP (Pan-Retinal Photocoagulation) treatment, etc. These small dark spots cannot be further discriminated by this approach since all candidate extractors used in [25] were designed for roughly extracting small dark spot objects. Therefore, if fundus images contains more complicated small dark spots, the performance of this approach will be deteriorated, as indicated by the results on DiaRetDB1 V2.1 database [29] reported in [25].

This paper proposes a novel automated method for MAs detection. The key idea is that, to preserve the MAs with different appearance, intensity and size as many as possible, we only reject the obvious non-MA objects, e.g., vessels and some background noises, with the gradient vector analysis, and all the remained small dark objects are taken as MA candidates which are further classified with a class imbalance classifier.

There are four main contributions in this work. Firstly, a new vessel removal algorithm is proposed based on the multi-scale log condition number computed from gradient vectors, which can remove most of the vessels and preserve most of the true MAs. Secondly, a candidate MAs localization algorithm is presented based on the second order directional derivatives in different directions, which can accurately localize the MAs’ centers. Thirdly, a new set of features are extracted for candidate MA classification, which can effectively discriminate the true MAs and the non-MAs. In addition, class-imbalance classification is introduced and analyzed in candidate MAs classification, which can effectively identify the true MAs from a large number of non-MAs.

The remainder of this paper is organized as follows. The proposed candidate MAs extraction and classification are described in detail in Section Methods. The two public available databases and the evaluation metrics used in this work are introduced in Section Databases and Evaluation Metrics. The experimental results are provided and analyzed in Section Results and Analysis. Finally, this paper is concluded in Section Conclusions.

## Methods

**Fig 2:**
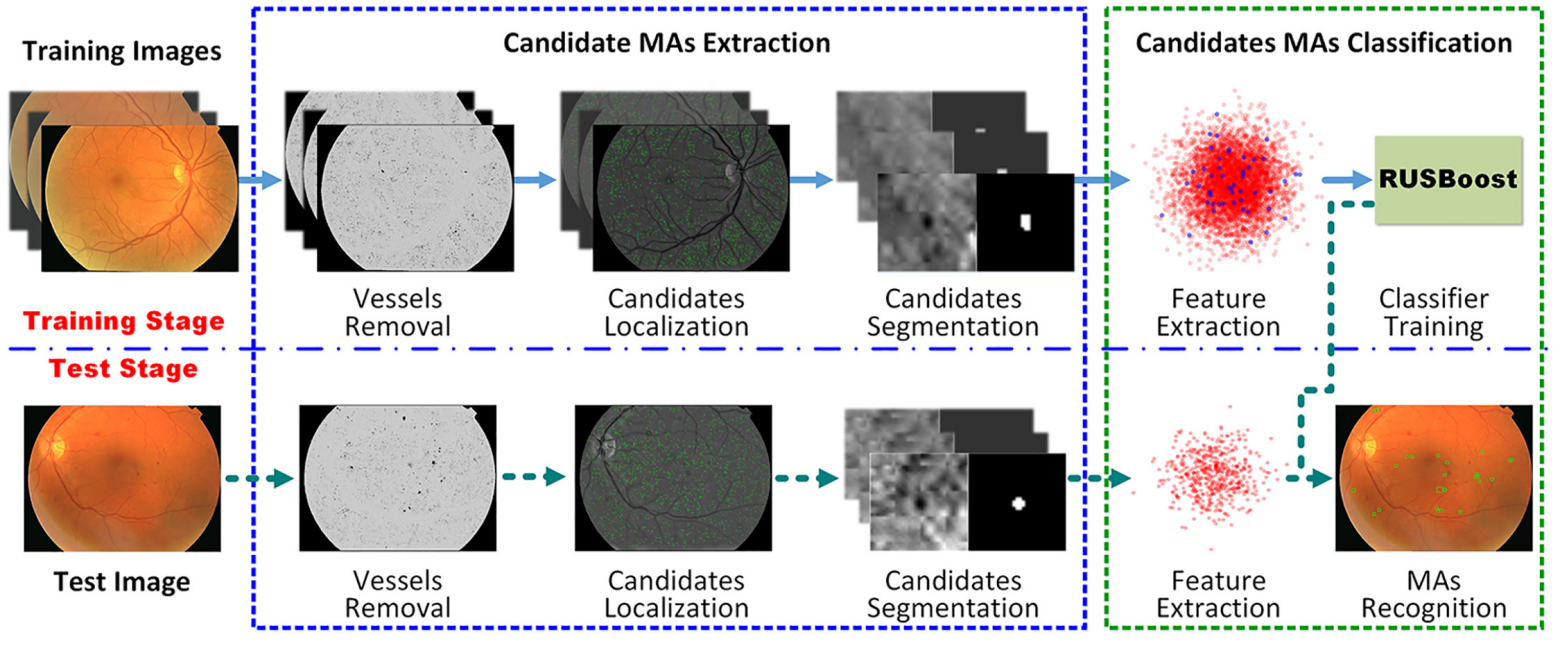
Schema of the proposed method. The solid line path summarizes the training process of MAs detection, and the dashed line path summarizes the test process.

As shown in Fig 2, the proposed MAs detection consists of two main steps, i.e., candidate MAs extraction and classification. In the candidate MAs extraction step, the main interferences, i.e., retinal vessels, are first suppressed, and then the candidate MAs are localized and segmented. In the candidate MAs classification step, a set of discriminative features are extracted and a class-imbalanced classifier is trained and used to identify the true MAs from amount of non-MAs.

### Candidate MAs Extraction

The proposed candidate MAs extraction includes vessel removal, candidate MAs localization and segmentation, and is described in detail as follows. As suggested in [18], the green channel of the color fundus image, where MAs have the highest contrast with the background, is used as the input image in this work. Before extracting candidate MAs, to smooth the image noises while preserving the boundary of MAs, an edge-preserving smoothing method [30] is first applied, which have been proved to be effective and can avoid introducing artifacts (e.g. ringing) that can deteriorate the performance of MAs detection. Next, a shade-correction method [26], which has been successfully employed in fluorescein image, is then used to reduce the uneven illumination of the smoothed input image. The final preprocessed image is denoted as *I*_*p*_.

#### Vessel Removal

**Fig 3:**
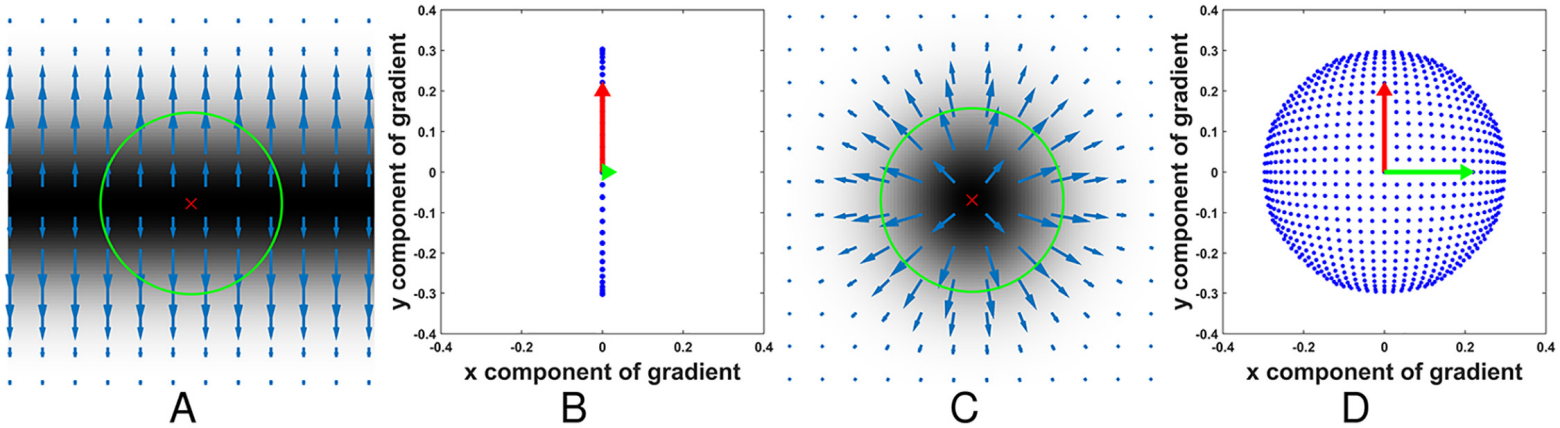
Different distributions of gradient vectors of the vessel-like and the MA-like objects. (A) The gradient field of a vessel-like object. (B) The distribution of gradient vectors in (A). (C) The gradient field of a MA-like object. (D) The distribution of gradient vectors in (C).

Because MAs are situated on capillaries and capillaries are not visible in color fundus images, they generally appear disconnected from the retinal vessel network [16]. Considering the vessels may affect MAs detection, we should remove vessels before extracting MA candidates. However, many existing vessel removal strategies may mistakenly remove some true MAs [18]. In this paper, we intend to remove vessels while preserving MAs with the gradient information. By observing the fundus images, we can find that retinal vessels always appear as piecewise linear structures, while the MAs generally appear as small round spots. Fig 3 shows the examples of these two structures and the distribution of their gradient vectors, where Fig 3A and 3C illustrate a linear structure and a round spot with their gradient vectors, and Fig 3B and 3D plot the distributions of the gradient vectors in Fig 3A and 3C. Let *S* denote a local support region, (*x*_*i*_, *y*_*i*_)(*i* = 1, …, *N*) denote all points in *S*, and (Δ*x*_*i*_,Δ*y*_*i*_) denote the gradient vector of the point (*x*_*i*_, *y*_*i*_). If *S* is a part of a vessel segment, which always can be regarded as a linear structure, the intensities always gradually increase from the centerline to the boundary along the perpendicular directions of this vessel (see Fig 3A), and the gradient vector directions (GVD) of most points on this vessel segment are perpendicular to the vessel segment (see Fig 3A). That is, the perpendicular direction of the vessel segment is the dominant principle direction of the distribution of (Δ*x*_*i*_,Δ*y*_*i*_) (see Fig 3B). While, if *S* located at the center of a true MA, which is always a blob-like structure, the intensities gradually increase from the center point to the boundary along the radial directions of this MA (see Fig 3C), and the GVD of the points in this MA are always similar with their corresponding radial directions. Since the radial directions of a blob can be arbitrary, no principle direction of the distribution of (Δ*x*_*i*_,Δ*y*_*i*_) is dominant (see Fig 3D). Therefore, we can determine if *S* is part of the retinal vessel or not by analyzing the principle direction of the distribution of (Δ*x*_*i*_,Δ*y*_*i*_). In this work, the covariance matrix of (Δ*x*_*i*_,Δ*y*_*i*_) are constructed and then the corresponding eigenvalues are computed to analyze the principle directions. For the vessel, there should be one dominant eigenvalue of the covariance matrix. While for the MAs, the two eigenvalues should be approximately equal.

More specifically, for each pixel (*x*, *y*) in the preprocessed image image *I*_*p*_, a circular support region with radius *r* centered at (*x*, *y*) is denoted as *S*_*r*_, and the covariance matrix of the gradient vectors in *S*_*r*_ is denoted as **C**(*x*, *y*, *r*). The eigenvalues of **C**(*x*, *y*, *r*) denotes as *λ*_1_ and *λ*_2_, and *λ*_1_ ≥ *λ*_2_.

The above-mentioned eigenvalue relations now can be reflected by the ratio *λ*_1_/*λ*_2_, i.e., the condition number of **C**(*x*, *y*, *r*), which is denoted as *κ*(**C**(*x*, *y*, *r*)). The condition number is closer to 1, the object is more circular, while the higher the value, the more elongated the object. This number thus gives a high discriminability between vessel-like and MA-like objects.

To deal with the size variation of both vessels and MAs in fundus image, we compute the condition number in a multi-scale manner by changing the radius of support region. A multi-scale log condition number K at (*x*, *y*) is defined by

$$\begin{array}{c} K\left(x,y\right)=\ln\prod_{r=r_{min}}^{r_{max}}\kappa \left(C\left(x,y,r\right)\right)=\sum_{r=r_{min}}^{r_{max}}\ln(\kappa (C(x,y,r))), \end{array}$$ (1)

where the logarithm function is used to prevent overflow. For our experimental data, the radius of most vessels and MAs is both generally varied from 1 to 6 pixels in the image with the smallest image width (768 pixels). We thus choose *r*_*min*_ = 2*ρ*, *r*_*max*_ = 7*ρ*, where *ρ* = (*image width*)/768.

**Fig 4:**
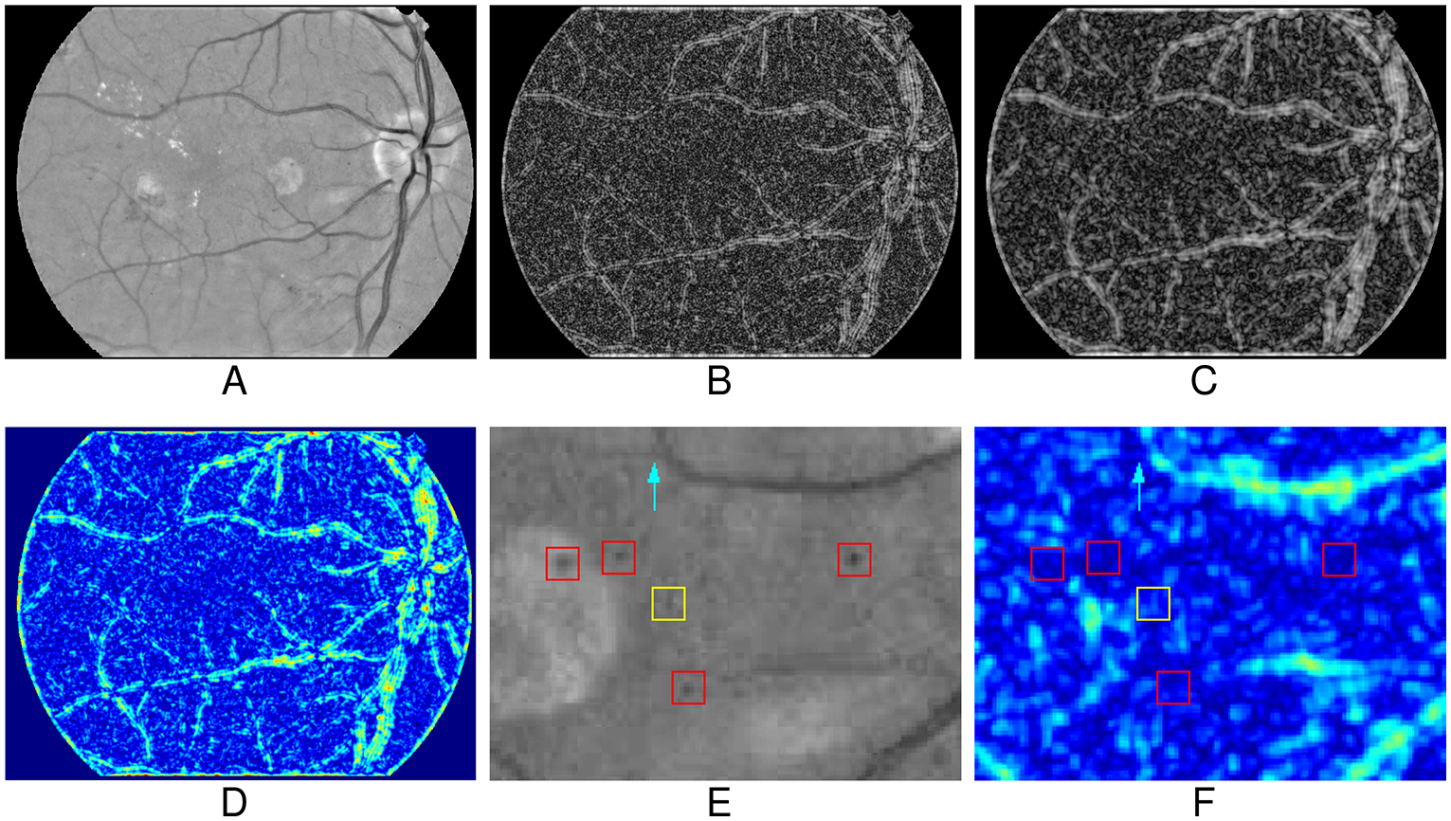
The process of K map computation. (A) The preprocessed input image. (B)-(C) The log condition number maps with different support regions. (D) The final K map. (E) An image patch of preprocessed image contains 5 true MAs marked with ‘□’. (F) The corresponding patch of K map.

Fig 4 shows the process of K map computation of the preprocessed image (Fig 4A). Fig 4B and 4C are the log condition number maps with respect to the support regions with different radius, and Fig 4D shows the final K map. As can be seen, most of the vessels, including the small and the large ones, have higher value of K. Fig 4E is a patch of the preprocessed image containing 5 true MAs marked with ‘□’, and Fig 4F is the corresponding patch of K map, from which we can see that all five true MAs of different size have lower responses than most of vessels. According to this figure, in K map, the vessel structures have been enhanced, while the MAs-like objects have been suppressed. We will separate vessels from MA-like objects according to the K map.

A similar principle for distinguishing the vessels and MAs was presented in [31], where the production and mean of the eigenvalues of Hessian matrix was exploited. Compared with the covariance matrix of a region centered at a pixel used in this work, the Hessian matrix of a pixel is more sensitive to noises. Unlike the proposed covariance matrix based method, the Hessian matrix based approach in [31] cannot use the information of a pixel’s surrounding pixels, which are very important to judge the pixel as a MA or vessel pixel. The estimation of Hessian with multi-scale Gaussian smoothing in [31] also easily impair the extraction of faint MAs and MAs near vessels. Additionally, the production and the mean of eigenvalues may not effectively distinguish the faint MAs (low contrast and small variety of intensity) with two small eigenvalues and the vessels with one large eigenvalue and one small eigenvalue.

From Fig 4E and 4F, since the MA marked with the yellow ‘□’ has an irregular shape, its response is slightly higher than the response of other MAs. And some vessel segments, e.g., the one indicated with the cyan arrow, also have lower K responses. Therefore it is difficult, if not impossible, to obtain the vessels map without MAs by directly applying threshold segmentation on K map.

**Fig 5:**
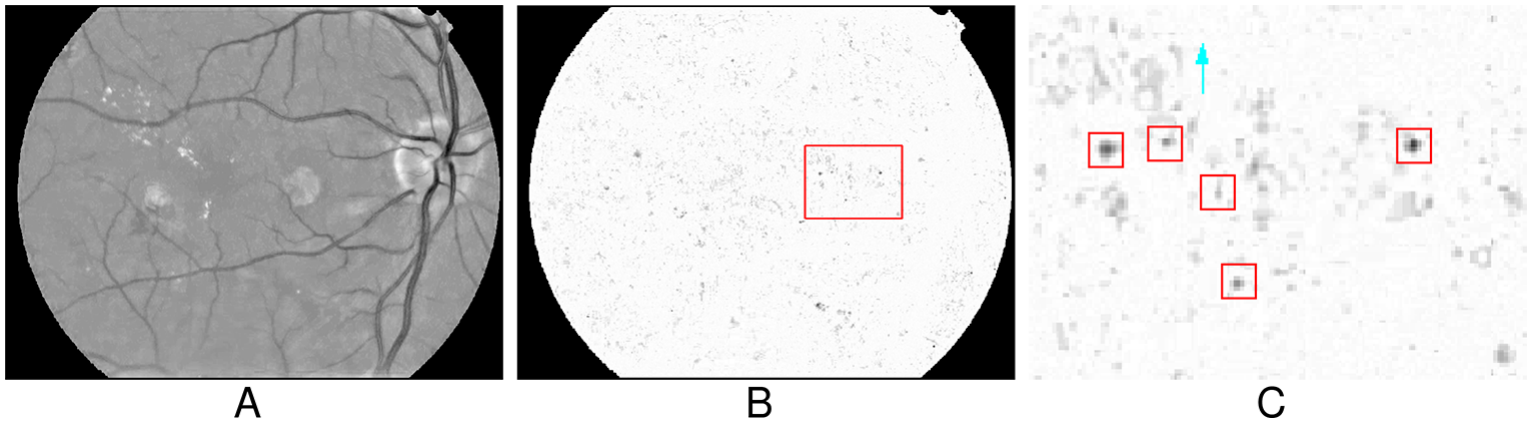
The process of vessel removal. (A) The image morphologically reconstructed by using a binary K map. (B) The vessel removed image by subtracting (A) from Fig 4A. (C) The enlarged part of marked region in (B).

**Fig 6:**
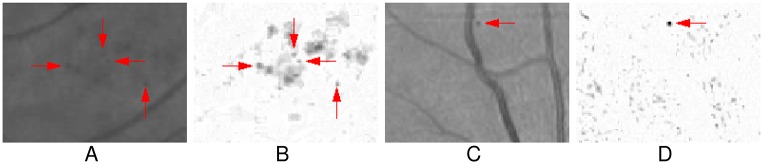
Some results of vessel removal in special cases. (A) The image patch containing subtle MAs with different size. (B) The corresponding result after vessel removed. (C) The image patch containing a MA near vessel. (D) The corresponding result after vessel removed.

In this work, we remove vessels based on the morphological grayscale reconstruction algorithm [32] with the K map. Fig 5 shows the process of vessel removal. An empirical threshold is firstly applied to K map to get a binary image, denoted as *I*_*bw*_, which includes the most vessel-like objects. The threshold value is set to 14.5 in this work according to the preliminary experiments on the training set. Then *I*_*bw*_ serves as a marker to reconstruct vessel map from the complement of the preprocessed image *I*_*p*_, denoted as *I*_*v*_ (Fig 5A). Since the marker *I*_*bw*_ only contains the rough vessel structures, only the vessel map can be morphologically reconstructed. Since MAs are not connected with vessels and not appeared in the marker *I*_*bw*_, MAs cannot be reconstructed. Thus, by algebraic subtracting *I*_*v*_ from *I*_*p*_, most vessel structures are removed efficiently, while the MAs-like objects with a variety of appearance, intensity and size are preserved, as shown in Fig 5B. Fig 5C is an enlarged part of the marked region in Fig 5B, which is also the corresponding vessel removed result of Fig 4E with the K map in Fig 4F. As shown in this figure, the vessel segments with low value of K are also removed, while the MAs with irregular shape are preserved successfully. Fig 6 also shows some results of vessel removal, in which the MAs, including the subtle ones (Fig 6A) and the one near vessel (Fig 6C), are successfully preserved.

In [17], the bottom-hat and hit-or-miss transformations were used to identify the MAs and vessels by considering the sizes of different structures in the fundus image. However, due to the size variety of both MAs and vessels, this approach may not work effectively. To extract candidate MAs, Séoud et al. [23] used the morphological gray reconstruction based on only the contrast information without considering the shape criterion, which may mistakenly extract many false MAs and miss some ture MAs.

#### Candidate MAs Localization

Besides the MAs, there also exists some tiny vessel segments, many dark background noises or other dark round objects in the vessel removed image. We need to localize the candidate MAs and, at the same time, suppress these non-MAs as many as possible. Considering that the intensities of some non-MAs may be similar to the intensities of some subtle MAs, it is not feasible to localize candidates only based on their intensities.

**Fig 7:**
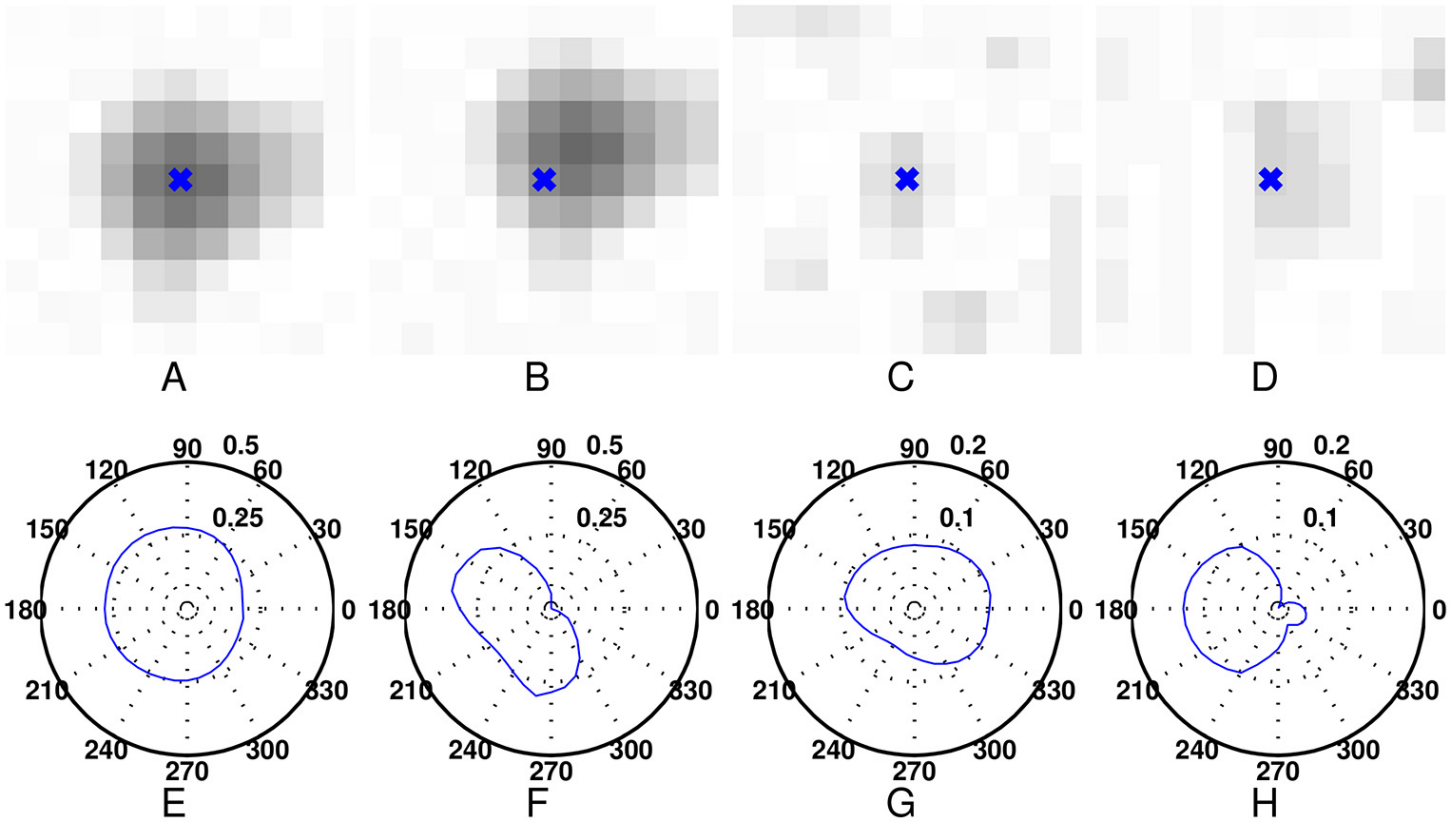
The illustration of $\tilde{I_{\theta }^{\prime \prime }}$ at different position with different direction *θ*. (A)-(B) The different positions of the same MA. (C)-(D) The positions centered at one subtle MA and one non-MA. (E)-(H) are the polar coordinate plots of the direction *θ* versus the value of $\tilde{I_{\theta }^{\prime \prime }}$ in the direction *θ* at these positions.

In general, MAs exhibit a Gaussian like intensity distribution in all directions [9, 10, 21]. According to this prior, Pereira et al. [33] used the gradient patterns and Gaussian fitting parameter in different directions to exclude the false MAs. In this work, we compute the second derivatives of grayscale profiles in different directions for MA localization, which can preserve the true MAs and exclude the false ones as many as possible, and at the same time, obtain more accurate MAs’ positions. Since MAs centers have the minimum intensity along 1-D grayscale profile in different directions, the center of MAs will have the positive local maximum of second derivatives of the grayscale profiles in all directions. While for the other positions of MAs or the positions of some non-MAs, the second derivatives in some directions will decrease or even be close to or less than zero. Fig 7 shows the distribution of second derivatives at different positions with different direction varied from 0° to 360° (all negative second derivatives are set to zero), where Fig 7A and 7B shows the different positions of the same MA, and Fig 7E and 7F are the polar coordinate plots of the direction versus the value of second derivative in the direction at these positions; Fig 7C and 7D shows the positions centered at one subtle MA and one non-MAs with the similar intensity as the subtle MA, and Fig 7G and 7H are the corresponding polar coordinate plots. From this figure, we can see that only the centers of MAs have high positive value of second derivatives in all directions, while other positions have low value of second derivatives close or equal to zero in some directions. Based on this observation, we localize the centers of the candidate MAs in vessel removed image with the second derivatives in multiple directions, i.e., the second directional derivatives [34], which can be computed by using gradient vectors.

Given a discrete image *I*, for each point (*x*, *y*), a continuous surface is first fitted using a facet model [34, 35] over the intensity values in a local window with a size of 7 × 7 centered at that point. The partial derivatives of the discrete intensity surface at (*x*, *y*) are then approximated by the corresponding partial derivatives of the continuous surface at that point [35]. Let’s consider a direction *θ* and the corresponding unit vector **u** = [cos*θ*, sin*θ*]^*T*^. The gradient vector of *I* here is denoted by $\nabla I={\left[\frac{\partial I}{\partial x},\frac{\partial I}{\partial y}\right]}^{T}$, and the directional derivative of *I* at the point (*x*, *y*) in the direction *θ*, denoted by $I_{\theta }^{\prime }\left(x,y\right)$, is given by

$$\begin{array}{c} I_{\theta }^{\prime }\left(x,y\right)=\frac{\partial I(x,y)}{\partial x}\cos\theta +\frac{\partial I(x,y)}{\partial y}\sin\theta =u^{T}\nabla I\left(x,y\right). \end{array}$$ (2)

The second directional derivative of *I* at the point (*x*, *y*) in the direction *θ*, denoted by $I_{\theta }^{\prime \prime }\left(x,y\right)$, is then computed as

$$\begin{array}{ccc} I_{\theta }^{\prime \prime } & = & \frac{\partial I_{\theta }^{\prime }}{\partial x}\cos\theta +\frac{\partial I_{\theta }^{\prime }}{\partial y}\sin\theta \\ & = & \frac{\partial^{2}I}{\partial x^{2}}\cos^{2}\theta +2\frac{\partial^{2}I}{\partial x\partial y}\cos\theta \sin\theta +\frac{\partial^{2}I}{\partial y^{2}}\sin^{2}\theta \\ & = & u^{T}\nabla I_{\theta }^{\prime }=u^{T}\nabla \left(u^{T}\nabla I\right). \end{array}$$ (3)

For exploiting the intensity distribution of MAs efficiently, we analyzed the intensity distribution by computing the directional derivatives from 0° to 360°. Considering that a negative $I_{\theta }^{\prime }$ representing the intensity value along the direction *θ* is monotonically decreasing, which is not consistent with the intensity distribution along the MA center to the boundary in that direction, we set the negative $I_{\theta }^{\prime }$ value to zero in the computation of second order directional derivatives in direction *θ*, and define a modification of $I_{\theta }^{\prime \prime }$, denoted as $\tilde{I_{\theta }^{\prime \prime }}$, as follows:

$$\begin{array}{c} \tilde{I_{\theta }^{\prime \prime }}=\frac{\partial \lfloor I_{\theta }^{\prime }\rfloor }{\partial x}\cos\theta +\frac{\partial \lfloor I_{\theta }^{\prime }\rfloor }{\partial y}\sin\theta =u^{T}\nabla \left\lfloor I_{\theta }^{\prime }\right\rfloor =u^{T}\nabla \left(\;\left\lfloor u^{T}\nabla I\right\rfloor \right), \end{array}$$ (4)

where ⌊⋅⌋ denotes that the enclosed quantity is equal to itself when its value is positive, and zero otherwise. These multiple second directional derivatives in different direction *θ* are then integrated by the following equation:

$$\begin{array}{c} P=\prod_{\text{all}\;\theta }\lfloor \tilde{I_{\theta }^{\prime \prime }}\rfloor =\prod_{\text{all}\;\theta }\lfloor u^{T}\nabla \left\lfloor I_{\theta }^{\prime }\right\rfloor \rfloor =\prod_{\text{all}\;\theta }\lfloor u^{T}\nabla \left(\left\lfloor u^{T}\nabla I\right\rfloor \right)\rfloor , \end{array}$$ (5)

Obviously, the center of MA-like objects can get much higher P values than other positions. In this work, we considered 36 directions from 0° to 360° with 10° step for P map computation.

**Fig 8:**
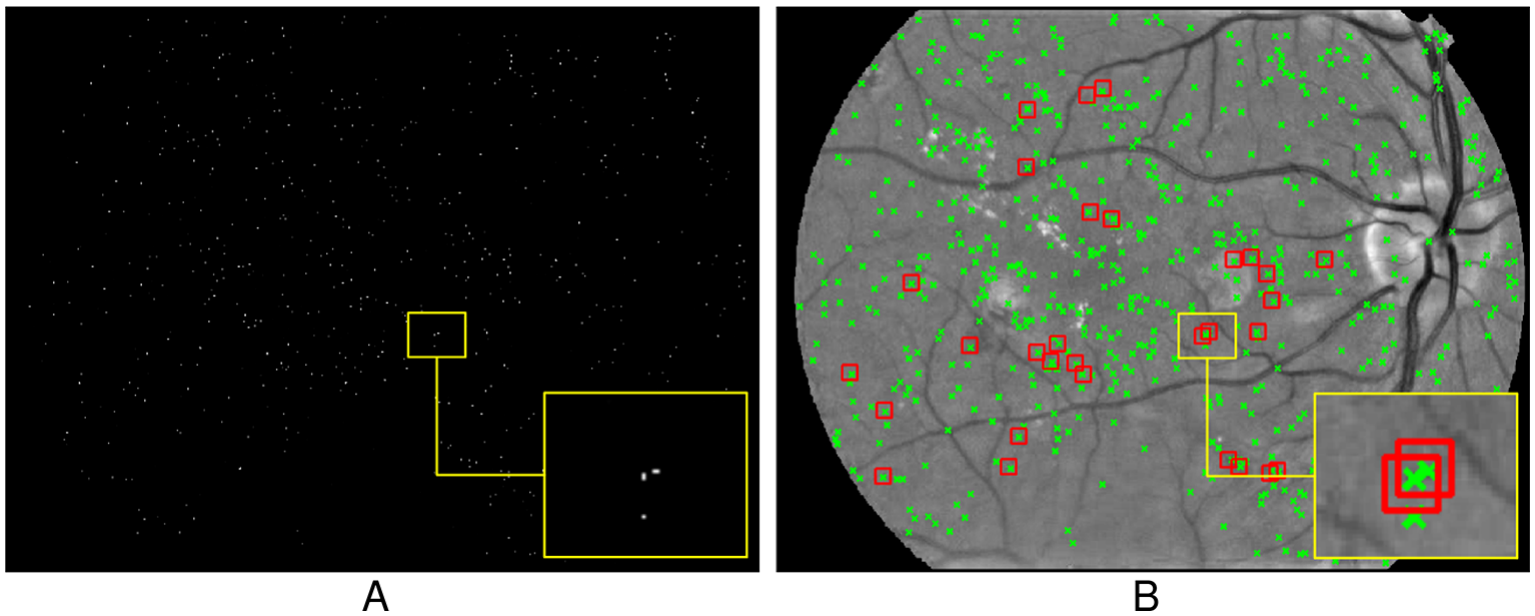
The result of candidate MAs localization. (A) The P map. (B) The final location of candidates MAs, where all candidates indicated with ‘×’ and the true MAs provided by medical expert marked with ‘□’.

Finally, we find all local maxima on the P map, and consider those whose P values are greater than a threshold as the final locations of candidates. The threshold here is empirically chose as the 0.1 times of the maximal P value. Fig 8 shows the results of candidate MAs localization of Fig 4A, in which Fig 8A is the P map, and Fig 8B is the final location of candidate MAs, indicated with the green ‘×’. As shown in this figure, the proposed technique can localize almost all of the true MAs (marked with ‘□’) annotated by medical experts. It should be noted that the MAs clustered together have also been localized separately, as shown in the enlarged patch of Fig 8, which often be treated as one candidate by other algorithms.

#### Candidate MAs Segmentation

After localizing all the MA candidates, the whole regions of these candidates should be segmented for following candidate MAs classification. To be robust to the variability of intensity across the fundus image, we adopt a localized-based level set model [36] to segment the whole regions of these candidates, in which a localized based Chan-Vese energy [36] is applied to drive the evolution of the level set. The localized based Chan-Vese energy is formed by replacing global means (of interior and exterior regions) in the original Chan-Vese energy with the means of the local regions of each active point. To improve the efficiency of the segmentation, we compute and update the values of level set in abovementioned model based on the sparse field technique [37], with which an efficient representation of level set can be maintained. The sparse field technique uses the linked-lists of the active points of the zero level set and their neighbor points to efficiently represent the level set, in which only the varying active points and their neighbors are updated.

**Fig 9:**
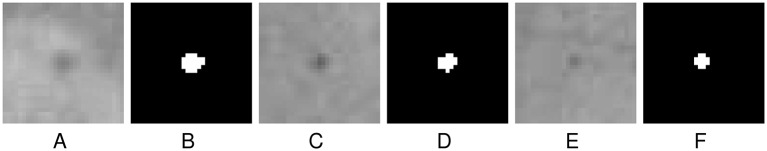
Segmentation results of candidate MAs. (A), (C) and (E) The image patches containing MAs. (B), (D) and (F) The segmentation results.

Fig 9 shows some results of segmentation, in which candidates with different size and various local contrast are successfully segmented.

### Candidate MAs Classification

#### Feature Extraction

To distinguish the true MAs and the non-MAs, this work extracts seven types of features for each candidate MA, i.e. geometric, contrast, intensity, edge, texture, region descriptors and other features. Excepting the region descriptors, the remaining extracted features (called common features) are commonly used to recognize the true MAs from the non-MAs based on the round shape and the color prior of true MAs, and most of them are adjusted from [10, 18, 22]. Region descriptors are introduced to exploit the local information of the candidate MA and its surrounding area for MAs classification.

1. *Geometric features:* The ratio *r*_1_ between the minor and the major axis length of the candidate region Ω. The ratio *r*_2_ between the diameter of a circle with the same area as Ω and its major axis length. The area *a*, the circularity *c*, the eccentricity *e* and the compactness *v* of Ω [18].
2. *Contrast features:* The intensity difference *ξ* between the maximum intensity value of the inside pixels and the minimum intensity value of the outside pixels of Ω in the preprocessed image *I*_*p*_ and each channel of the RGB, LUV, and HSI color spaces of the original color fundus image *I*_*o*_. Notice that the outer region of Ω here is the region obtained by removing Ω from its morphological dilated version.
3. *Intensity features:* The total intensity Σ of Ω, the normalized intensity *ni* and the normalized mean intensity *nm* in *I*_*p*_ and the green channel of *I*_*o*_ [18]. The mean intensities *μ*_*in*_, *μ*_*out*_ and their corresponding standard deviations *σ*_*in*_, *σ*_*out*_ of the inside pixels and the outside pixels of Ω in each channel of the RGB, LUV, HSI color spaces of *I*_*o*_.
4. *Edge features:* The mean value *μ*_*e*_ of the gradient magnitude of pixels on the boundary of Ω in *I*_*p*_.
5. *Texture features:* The mean value *μ*_*g*_ (*μ*_*l*_) and the standard deviation *σ*_*g*_ (*σ*_*l*_) of Ω in the Gaussian (LoG) filter responses of *I*_*p*_ with *σ* = 1, 2, 4, and 8 [10].
6. *Region descriptors:* The region descriptors, i.e. HOG [38], SURF [39] and GIST [40] descriptors, of the local image patch centered at Ω in *I*_*p*_. Since the radius of the manually labeled MA mask is commonly between 5∼10 pixels, to include the information of both the candidate MA region and its surrounding region in the region descriptors, the patch size is set to 31 × 31 pixels (about a radius of 15 pixels). The HOG descriptor computes locally normalized histograms of gradient orientation in 5 × 5 grids for the local patch, in which each cell have 31 features. The SURF descriptor computes 8 features about Haar wavelet responses for each cell of the 4 × 4 grids of the patch. The GIST descriptor utilizes the Gabor filter responses in 3 scales and 8 orientations in 3 × 3 grids to provide a rough representation of the patch. The total amount of HOG, SURF and GIST features are 775, 128 and 216 respectively.
7. *Other features:* The mean value *μ*_*ξ*_ and the standard deviation *σ*_*ξ*_ of the angle differences between the gradient vector and the unit vector along the different sampling directions within Ω computed in *I*_*p*_ [22]. The mean values *μ*_*κ*_, *μ*_*ci*_ and *μ*_*div*_ and the standard deviation *σ*_*κ*_, *σ*_*ci*_ and *σ*_*div*_ of the inside pixels and the outside pixels of Ω in the condition number map, the convergence index map [41] and the divergence map [42] computed in *I*_*p*_. The mean value *μ*_*ii*_ and the standard deviation *σ*_*ii*_ of Ω in the isolated index map, which is the ratio of the mean intensity to the standard deviation in a ring region with width of 3 pixels outside a circular support region with radius of 7 computed in *I*_*p*_. The mean value *μ*_*wi*_ of candidate pixels in the product image of condition number, convergence index, isolated index and divergence maps.

In summary, the total feature set contains 1247 features. Since those region descriptors are seldom applied in candidate MAs classification, we will investigate their contributions for identifying MAs in Section Results and Analysis.

#### MAs Recognition

After extracting features for candidates, the true MAs should be identified in these candidates. However, as mentioned previously, in the extracted candidate MAs, the non-MAs are much more than the true MAs. In this work, since we intend to preserve more MAs in the extraction stage, the ratio is much higher, which can be close to 500:1, as shown in Section Introduction.

To address this issue, a simple exclusion criterion is firstly used to remove some obvious false positives according to the discrimination table in [10] and our preliminary experiments, such as the candidates whose area *a* are out of the range from 2 to 150 or whose ratio *r*_1_ are less than 0.3. And then a class-imbalance classifier is trained to recognize the true MAs.

The RUSBoost learning algorithm [43], which embedded the technique of random undersampling into the AdaBoost.M2 algorithm [44], has been previously shown to be very effective at alleviating the problem of class imbalance. Hence a RUSBoost classifier is trained to recognize the true MAs from a large number of non-MAs in this work.

Given the minority training set *R* and the majority training set *S*, where |*R*| ≪ |*S*|, the RUSBoost randomly undersamples a subset *S*′ from *S* in each iteration of AdaBoost.M2, and construct the temporary class-balanced training set *R* ∪ *S*′ to train weak learners, where |*S*′| < |*S*|, and usually, |*S*′| = |*R*|. The final strong classifier *H*(*x*) is a weighted combination of *T* weak classifier *h*_*t*_ (*t* = 1, 2, …, *T*), which are trained with class-balanced subset $R\cup S_{t}^{\prime }$ instead of *R* ∪ *S* in round *t*.

## Databases and Evaluation Metrics

The proposed method is evaluated on two public database, i.e., the Retinopathy Online Challenge (ROC) competition [3] and DiaRetDB1 V2.1 database [29].

### The ROC database

The ROC database consists of 100 images with different resolutions [3], which are randomly split into a training and a test set, each containing 50 images. ROC only provide the ground truth of training set, which contains the location and the radius of each manually labeled MA. The proposed method is trained on the training set, and the ROC organizer evaluates it on the test set.

### The DiaRetDB1 V2.1 database

The DiaRetDB1 V2.1 (denoted as DRDB) database contains 89 color fundus images with the fixed 1500 × 1152 resolution [29]. Among these fundus images, 28 images are given for training and the remaining 61 images are for testing. For each image in both the training and the test set, four ground truths annotated by different experts are provided, which include the location, the radius, and the confidence level of each marked MA.

### Evaluation Metrics

In this work, a finding is defined as a “hit” of a manually labeled MA if the MA is the closest one to the finding and the distance between the center of the finding and the center of the MA is smaller than the provided radius. A finding is defined as a “hit-miss” if the distance between the center of the finding and the center of any manually labeled MA is larger than the provided radius. One marked MA may have multiple “hits”, but we only count a single “hit” of each MA as a *true positive* (TP), and count the other “hit” of the same MA as a *false positive* (FP), as defined in the ROC competition [3]. All “hit-miss” are also counted as the false positives. The sensitivity is then computed as #TPs/#T_*true*_, where #TPs is the number of the true positives, and #T_*true*_ is the number of the manually labeled MAs. An average number of false positives per image (FPs/I) is also used to analyze the sensitivity in the evaluation.

For fairly evaluating different methods, the ROC competition organizer do not provide the ground truth of the ROC test dataset to avoid training parameters on this dataset. Therefore, we cannot compare our output results with the ground truth images to get the sensitivity and specificity on this dataset. The detected MAs of different methods are submitted to the ROC organizer, and the organizer computes and returns the sensitivities at some levels of FPs/I and the competition performance metric (CPM, an average sensitivity at a set of particular false positives per image 1/8, 1/4, 1/2, 1, 2, 4 and 8 FPs/I) [3], which are used as the evaluation of different methods on the ROC test dataset. We thus evaluate the performance of the proposed method with these metrics on both ROC and DRDB database. Besides these metrics, we also evaluate our method with the free-response receiver operating characteristic (FROC) curve [3] and the partial area under the curve (AUC) of the FROC curve [25]. The FROC curve plots the sensitivity against the number of FPs/I. The partial AUC is the partial area under the FROC curve between 1/8 and 8 FPs/I.

## Results and Analysis

### Evaluation of Candidate MAs Extraction

Table 1 compares the sensitivities of the proposed candidate extractor and the state-of-the-art candidate extractor algorithms [10, 16, 26–28] with the same level of FPs/I on the ROC training set, where our results are listed in the columns titled “Our Sen.”. The results of state-of-the-art approaches are reported in [25] and [45]. As Table 1 demonstrated, the proposed method notably improves the sensitivities of candidate MAs extraction. Although sensitivity is slightly lower than Lazar et al. [28] at the level of 73.94 FPs/I, our method achieves much higher sensitivity at the level of 569.39 FPs/I. Therefore, the proposed candidate extractor can characterize MAs better than other approaches.

**Table 1:** Comparison of different candidate extractors on ROC training set.

Method	FPs/I	Sen.	Ours Sen.
Spencer *et al*. [26]	20.30	0.12	**0.29**
Lazar et al. [28]	73.94	**0.48**	0.47
Walter *et al*. [16]	154.42	0.36	**0.55**
Zhang *et al*. [10]	328.30	0.33	**0.62**
Abdelazeem [27]	505.85	0.28	**0.68**
Lazar et al. [28]	569.39	0.598	**0.691**

After filtering out some obvious non-MAs from the candidate MAs, the remaining candidates form the final candidates set for the following classification. When a manually labeled MA has multiple “hits”, we cannot tell apart which one is true. To avoid losing the positive samples in the candidate MAs classification stage, we take all of the “hits” as positive ones (called positive candidates) and all of the “hit-miss” as negative ones (called negative candidates) when training the classifier. Table 2 lists the number of positive candidates (#PCs), the number of negative candidates (#NCs), and the ratio between #NCs and #PCs (Ratio; referred to the level of imbalance) of the final candidates sets extracted from the training set of different databases. In particular, we list all results of DRDB database with different ground truths in this table. Since the experts have different clinical experiences, the ground truths annotated based on their experiences in DRDB database vary largely, which result in the large variance of the statistic results in Table 2. Especially, for the ground truth annotated by the Expert 2, the marked areas are much larger than those marked by other experts, therefore much more candidates fall into these areas. It is the reason that the #PCs with Expert 2 are much larger and the Ratio is much smaller than those with other experts. According to this Table, the ratios between the negative candidates and the positive candidates are very high, the candidate MAs classification is thus an extremely class imbalanced problem in this work.

**Table 2:** The statistical results of final candidate sets extracted from training set of different databases.

Training set	#Images	#PCs	#NCs	Ratio
ROC	50	284	123042	433:1
DRDB w/ Exp. 1	28	317	81734	258:1
DRDB w/ Exp. 2	28	4424	77627	18:1
DRDB w/ Exp. 3	28	162	81889	505:1
DRDB w/ Exp. 4	28	314	81737	260:1

### Evaluation of Class Imbalanced Candidates Classification

**Fig 10:**
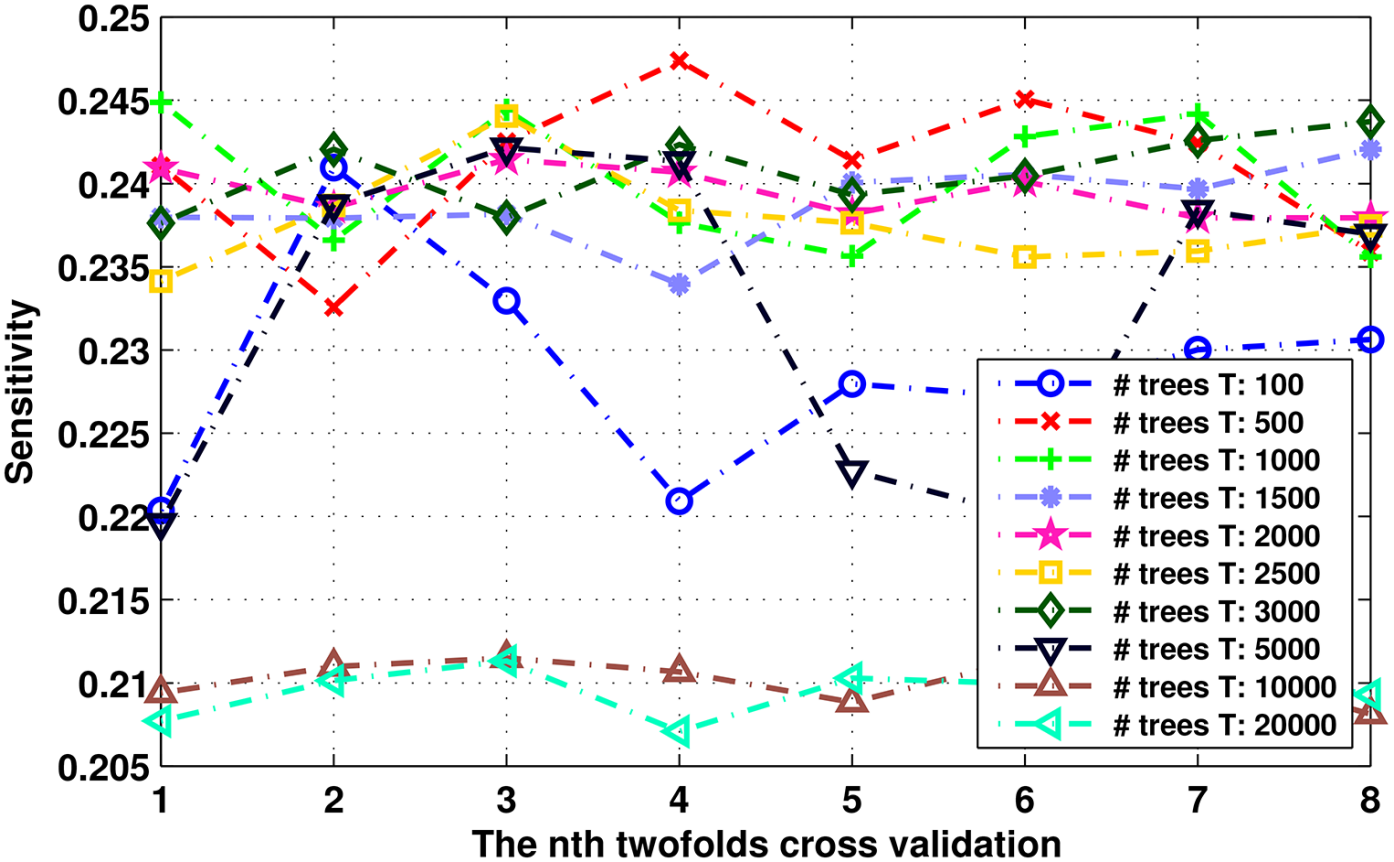
The sensitivities of cross validation on ROC training set with different *T* in RUSBoost classifier.

In our experiments, we use the decision trees as the weak learners in the RUSBoost model. To get the optimal number of weak learners *T* used in RUSBoost, we randomly split the ROC training set into two subsets, and perform 8 times two-folds cross validation for different *T*. Fig 10 shows the performance of each round of twofold cross validation on the ROC training set with the different number *T* in the RUSBoost. The classifier achieves a relatively stable sensitivity of 0.24 ± 0.01 when *T* is about 500 to 3000, and the overfitting occurs when *T* > 3000. We set *T* = 500 trees in this work to test the proposed method on both ROC and DRDB database.

**Fig 11:**
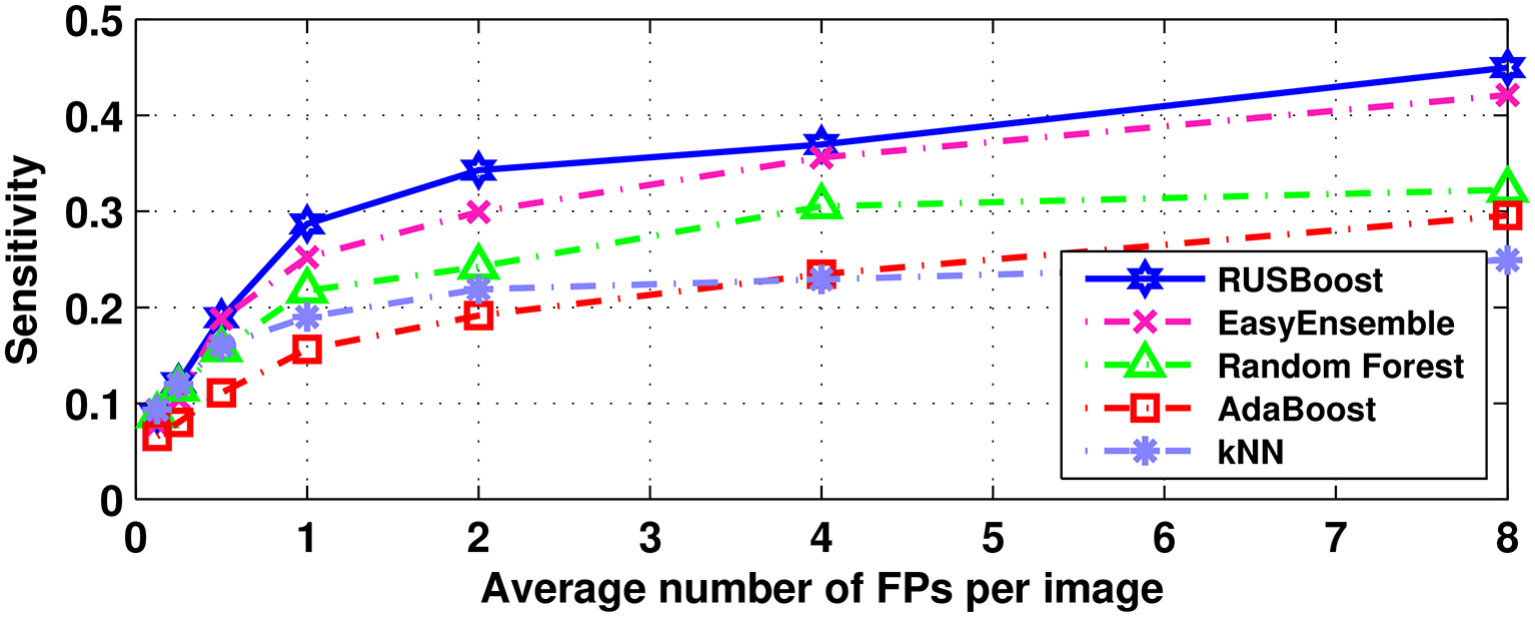
FROC curves produced by different classifiers.

To evaluate the performance of class imbalance classifier in MAs detection, we choose three ensemble classifiers, i.e., RUSBoost [43], EasyEnsemble [24] and AdaBoost [44], where the first two classifiers are especially designed for class-imbalance learning. We also test the *k*NN classifier, which has been successfully used for MAs classification in [14, 18, 46]. The performance of a Random Forest classifier is also tested, which has been used in [23] to recognize true MAs from class-imbalanced data. The FROC curves of these classifiers on ROC training set are plotted in Fig 11. Since the samples are extremely imbalanced, it is not surprised that the class imbalance classifiers notably outperform the other classifiers in MAs classification. Although the Random Forest has slightly improved the performance of *k*NN and AdaBoost for class-imbalanced problem, the RUSBoost, which integrated a under-sampling and boosting techniques, obtained the best result and improved the classification performance significantly.

### Evaluation of the Overall MAs Detection

We next test the overall performance of MAs detection with the proposed method on both ROC and DRDB database.

**Fig 12:**
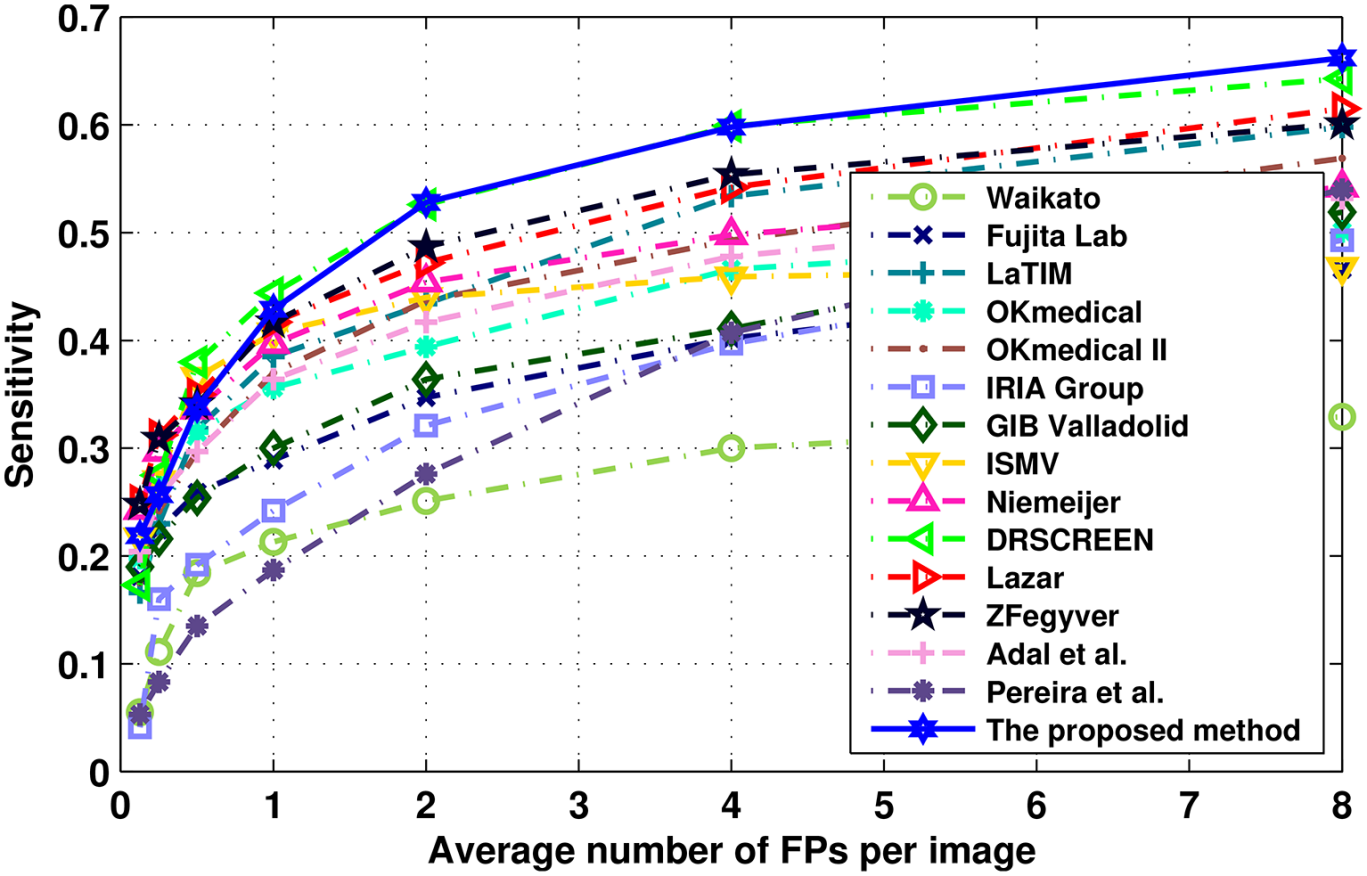
The FROC curves for each of different approaches on the test set of the ROC competition.

Table 3 lists the sensitivities at the predefined FPs/I, the ranked CPM evaluated by ROC organizer, and the partial AUC of 15 participating teams of ROC. The proposed method was ranked in the second place among these approaches with the score of 0.433, which is very close to the score of 0.434 of the first placed ensemble approach DRSCREEN [25]. And the proposed method obtains the highest partial AUC (0.553) among all of these approaches. The FROC of these approaches are plotted in Fig 12, from which, we can see that the proposed method performs very well especially after the level of 2 FPs/I. According to Table 3 and Fig 12, the proposed method can get a comparable performance with the DRSCREEN approach.

**Table 3:** Quantitative results of the ROC competition for each participating team.

Team Name	1/8	1/4	1/2	1	2	4	8	CPM	AUC
DRSCREEN [25]	0.173	0.275	**0.380**	**0.444**	0.526	**0.599**	0.643	**0.434**	0.551
Our method	0.219	0.257	0.338	0.429	**0.528**	0.598	**0.662**	0.433	**0.553**
Lazar [21]	**0.251**	**0.312**	0.350	0.417	0.472	0.542	0.615	0.423	0.510
Fegyver [22]	0.248	0.309	0.341	0.417	0.487	0.554	0.601	0.422	0.514
Niemeijer [18]	0.243	0.297	0.336	0.397	0.454	0.498	0.542	0.395	0.469
LaTIM [9]	0.166	0.230	0.318	0.385	0.434	0.534	0.598	0.381	0.489
ISMV [20]	0.217	0.270	0.366	0.407	0.440	0.459	0.468	0.375	0.435
OKmedical II [47]	0.175	0.242	0.297	0.370	0.437	0.493	0.569	0.369	0.465
Adal et al. [46]	0.204	0.255	0.297	0.364	0.417	0.478	0.532	0.364	0.446
OKmedical [10]	0.198	0.265	0.315	0.356	0.394	0.466	0.501	0.357	0.430
GIB Valladolid [48]	0.190	0.216	0.254	0.300	0.364	0.411	0.519	0.322	0.399
Fujita Lab [49]	0.181	0.224	0.259	0.289	0.347	0.402	0.466	0.310	0.378
IRIA Group [15]	0.041	0.160	0.192	0.242	0.321	0.397	0.493	0.264	0.368
Pereira et al. [33]	0.053	0.083	0.135	0.187	0.276	0.407	0.540	0.240	0.366
Waikato [50]	0.055	0.111	0.184	0.213	0.251	0.300	0.329	0.206	0.273

Recently, Soares et al. [23] reported a sensitivity of 0.47 at 37.8 FPs/I on the training set of ROC database, while our method achieved a sensitivity of 0.49 at the same FPs/I on the same set.

**Fig 13:**
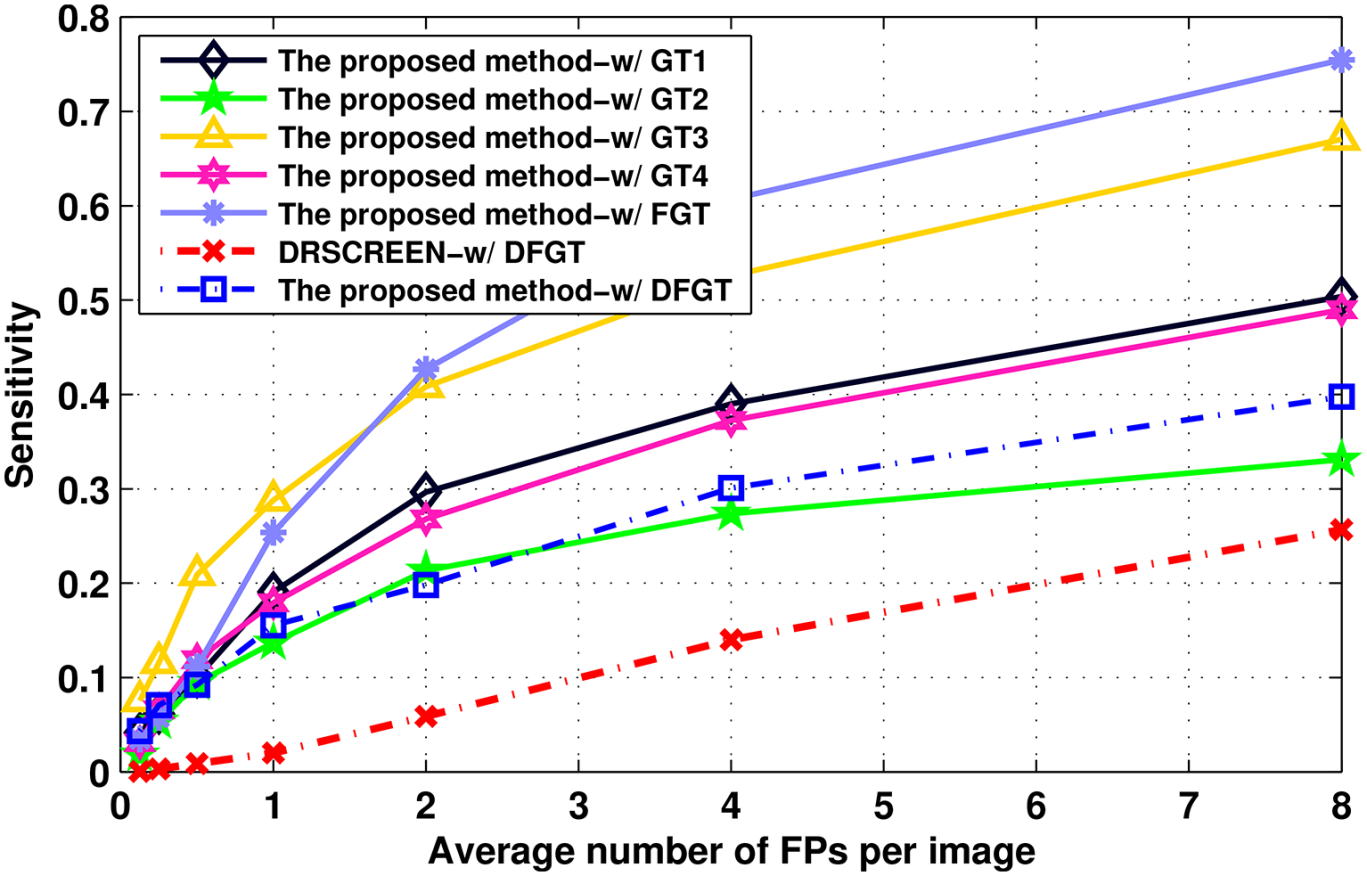
FROC curves of the proposed method and the DRSCREEN approach on the DRDB database. The FROC curve reproduced from the original work of DRSCREEN [25] on the same database.

DRDB database provides four ground truths annotated by different experts, respectively denoted as GT1, GT2, GT3 and GT4. Since there are disagreements among four experts’ annotations, we take a consensus of 75% agreement as the fusion ground truth, denoted as FGT. The proposed method is evaluated by using these ground truths. For comparison, a fused ground truth used by the DRSCREEN approach [25] (denoted as DFGT), provided by the authors, is also employed for evaluation. The FROCs of the proposed method with different ground truths and the FROC of DRSCREEN with DFGT, reproduced from the original work in [25] with the kind support from the authors, are plotted in Fig 13 and the sensitivities at the predefined FPs/I, the CPM scores and the partial AUC of the proposed method with different ground truths, and the results of DRSCREEN [25] with DFGT are listed in Table 4. According to this figure and table, the proposed method outperforms the DRSCREEN approach at all predefined FPs/I with all ground truths. Particularly, the CPM and partial AUC of the proposed method (0.180 and 0.270) are much higher than the those of DRSCREEN (0.070 and 0.130) with DFGT. The possible reason is that the small dark objects in DRDB database are more complicated, which contains not only MAs but also some small hemorrhages and scars left after PRP treatment. However, DRSCREEN only selects the optimal combination of several different preprocessing operations and several different candidate extractors, which are designed to extract small dark objects from fundus images, without further discriminating them. Therefore, DRSCREEN cannot discriminate these different small dark objects. While in this work, after extracting candidates, we have trained a classifier with a set of features for classifying these different small dark objects. Therefore, the proposed method can outperform the DRSCREEN approach [25] on DRDB database.

**Table 4:** The results of the proposed method and the DRSCREEN approach on the DRDB database.

Method	1/8	1/4	1/2	1	2	4	8	CPM	AUC
Ours w/ DFGT	0.043	0.071	0.092	0.155	0.198	0.301	0.398	0.180	0.270
DRSCREEN	0.001	0.003	0.009	0.020	0.059	0.140	0.257	0.070	0.130
Ours w/ GT1	0.042	0.061	0.100	0.191	0.296	0.390	0.504	0.226	0.352
Ours w/ GT2	0.018	0.053	0.094	0.137	0.213	0.274	0.331	0.160	0.244
Ours w/ GT3	**0.075**	**0.116**	**0.209**	**0.288**	0.408	0.526	0.671	**0.328**	0.482
Ours w/ GT4	0.031	0.066	0.119	0.179	0.268	0.372	0.490	0.218	0.336
Ours w/ FGT	0.035	0.058	0.112	0.254	**0.427**	**0.607**	**0.755**	0.321	**0.527**

Please note that the results of the proposed method evaluated with GT2 are much worse than those of the other ground truths (See Table 4). The reason is that the regions annotated by Expert 2 are too large and hence the label information of samples are too ambiguous, which result in much noise samples in classification and degenerate the performance.

Zhang et al. [10] reported an average sensitivities of 0.713 at three levels of FPs/I (i.e. 1/2, 1, 2) on the DRDB database with the FGT. It is much higher than the value of 0.264 of our method at those levels, but that approach only used 11 images randomly selected from the database for both training and testing purpose. Noted that our method was trained with the training set of 28 images and tested on the whole test set of 61 images. Recently, Adal et al. [46] achieved a sensitivity of 0.6462 at 10 FPs/I on the DRDB database with the FGT, while our method have achieved a higher sensitivity of 0.7753 at the same FPs/I. Rosas-Romero et al. [17] reported a sensitivity of 0.9232 at the specificity of 0.9387 on the DRDB database, which is tested on the both training and test set with a selected subset of 88 images. Noted that there is no description of the selection of the ground truth and the training set in [17]. We here trained our classifier with the training set of 28 images, and tested it on the subset of 88 images as used in [17] with the FGT. We achieved a sensitivity of 0.9337 at the same specificity. These improvements also demonstrates the effectiveness of our method in MAs detection.

### Evaluation of Region Descriptors in Classification

To investigate the contribution of region descriptors for candidates classification, we test the performances of different combinations of region descriptors and the common features. Table 5 shows the classification results on ROC training set with different feature combinations, i.e., without (w/o), only, or with (w/) the HOG (H), SURF (S) and GIST (G) features. According to this table, the common features achieve a CPM score of 0.235, while only the region descriptors can also achieve a score of 0.206. Although the score of the region descriptors is inferior to that of the common features, the region descriptors can achieve higher sensitivities than the common ones at the low FPs/I of 1/8 and 1/4, and the feature sets combined common features and region descriptors have improved the overall performance of candidate classification. More specially, the combination included common features and all three region descriptors have achieved the highest CPM score of 0.264. It is also noteworthy that the improvement of the combinations with HOG features are more significant than those without HOG, such as the score of ‘w/ H’, ‘w/ H+S’ and ‘w/H+G’ is 0.261, 0.263 and 0.262 respectively. The possible reason is that the HOG features are extracted in dense overlapping grids, such that they may provide more supplementary information than other descriptors for common features in MAs classification.

**Table 5:** Classification results of different feature combinations on ROC training set.

Features	1/8	1/4	1/2	1	2	4	8	CPM
w/o H+S+G	0.044	0.079	0.180	0.252	0.298	0.365	0.428	0.235
only H+S+G	0.078	0.107	0.139	0.226	0.240	0.296	0.356	0.206
w/ H	0.079	0.111	0.184	0.255	0.349	0.404	0.445	0.261
w/ S	0.043	0.076	0.174	0.245	0.338	0.399	0.438	0.245
w/ G	0.064	0.092	0.164	0.243	0.346	0.392	0.434	0.248
w/ H+S	**0.093**	0.121	0.181	0.259	0.349	0.397	0.440	0.263
w/ H+G	0.087	0.110	0.170	0.264	**0.353**	**0.405**	0.444	0.262
w/ S+G	0.087	0.088	0.154	0.278	0.345	0.398	0.435	0.255
w/ H+S+G	0.088	**0.122**	**0.189**	**0.287**	0.343	0.370	**0.450**	**0.264**

### Analysis

There are some errors of MAs detection with the proposed method in the experiments.

**Fig 14:**
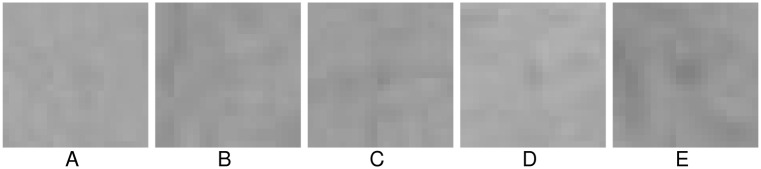
Examples of error detection. (A)-(B) The true MAs with low contrast. (C) The vessel crossing with high contrast. (D)-(E) The non-MAs have very similar appearance as true MAs.

Some true MAs with low contrast are not detected, as shown in Fig 14A and 14B. It is mainly because that the images in the databases are provided with a compressed format, by which these subtle MAs are heavily blurred and cannot be found out even by the naked eyes.

Some non-MAs were mistakenly detected as MAs, as shown in Fig 14C–14E. Fig 14C is a vessel crossing remained after vessel removal which is misclassified as MAs in classification stage. This is due to that these non-MAs have similar distribution of gradient vectors with the true MAs and have been extracted as candidates at the candidate MAs extraction stage, and the extracted features cannot discriminate these false positives at the classification stage. Fig 14D and 14E lists some isolated spots which are mistakenly detected as MAs. These non-MAs appeared almost identical to the true MAs, which are challenging to be distinguished even by ophthalmologists.

## Conclusions

This paper proposed a novel automated method for MAs detection in color fundus images, which contains two stages, i.e. candidate MAs extraction and classification. In the first stage, the vessels can be effectively removed and the candidate MAs can be accurately localized by analyzing the gradient vectors of the images, which demonstrates that the gradient vectors can reflect the characteristic of different objects on fundus images. Most of the true MAs can be effectively extracted in this stage. To classify the non-MAs and the true MAs, seven types of features, i.e. geometry, contrast, intensive, edge, texture, region descriptors and other features, are extracted in the second stage. These features, especially the region descriptors, can well characterize the true MAs and have high discriminability to the true and the non-MAs. Since the non-MAs and the true MAs are extremely class imbalanced in the extracted candidates, the class imbalanced classifiers, e.g. RUSBoost, can effective classify these candidates. The proposed method performs well on different databases.
